# Supplementary material for: Genes regulating membrane-associated E-cadherin and proliferation in adenomatous polyposis coli mutant colon cancer cells: High content siRNA screen
Source: PLoS One. 2020 Oct 15;15(10):e0240746. doi: 10.1371/journal.pone.0240746 (PMC7561197; doi:10.1371/journal.pone.0240746)

S1 Fig. Screening process & image analysis.

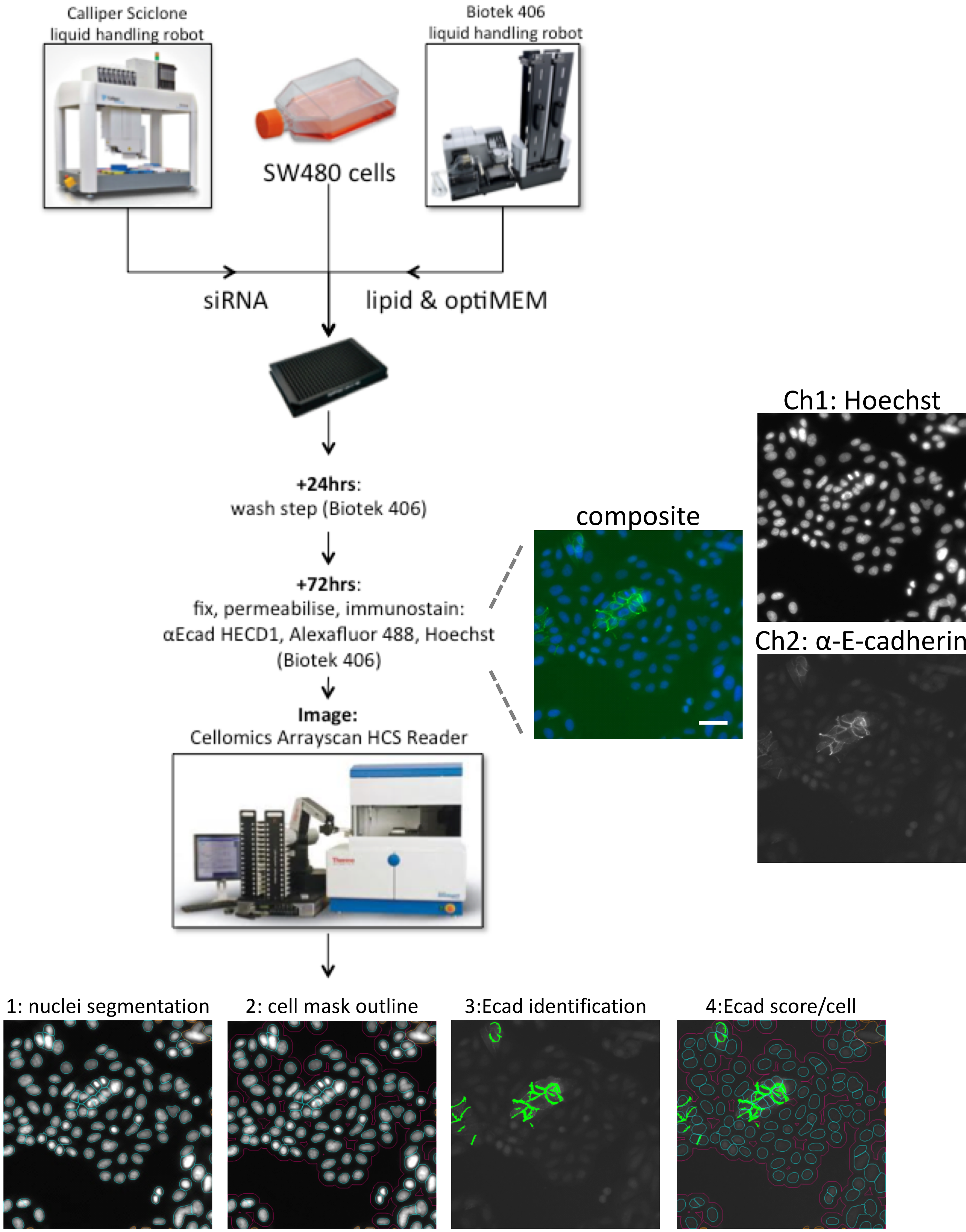

Supplement: S1 Fig — SW480 cells were thawed from liquid nitrogen and passaged so that for each round of transfections the cells were transfected at passage 5 and at day 8 post thaw. SW480 cells were reverse transfected using lipofectamine 2000 and 40nM of SMARTpool siRNA (Dharmacon RNAi Technologies) for the primary screen, and 25nM of the individual duplex siRNA in the deconvolution screen. The siRNA library (RefSeq v.27) was accessed through, and the screen performed at, the Victorian Centre for Functional Genomics (VCFG), Peter MacCallum Cancer Centre. Transfections took place in optical, black walled 384-well plates. All transfections and wash steps were carried out using the Caliper Sciclone ALH3000 and BioTek 406 liquid handling robots available at the VCFG. Cells were washed with 40μl of PBS and replaced with 40μl of fresh media 24 hours post transfection (medium flow rate). At 72 post transfection, the cells were fixed in 4% PFA/PBS, permeabilised, immunostained with αE-cadherin (HECD1) then Alexafluor-488 αmouse antibodies and co-stained with Hoechst 34442 to mark the nuclei. The plates were then imaged on the Cellomics ArrayScan VTi HCS Reader at 20X magnification using the Cellomics Morphology V.4 Bioapplication (see S1vi Table for algorithm settings). Briefly cells were identified in channel 1 using Hoechst stain. Identification of cells allowed the algorithm to identify cell number. This count is important for cell health, proliferation and toxicity reports, and to quantify E-cadherin levels (1). The algorithm created a ring around the nuclei edge. The ring was expanded away from the cell nucleus to identify a whole cell mask for each cell. The whole cell mask is required to quantify E-cadherin (2). E-cadherin staining was identified in channel 2 using the ‘fibre detection’ algorithm. Briefly the algorithm parameters were set to detect long fibre-like αEcad staining (3). The Ecad score was defined as the quantity of all E-cadherin fibres from channel 2 within the modified [file pone.0240746.s001.pdf]
