# Supplementary material for: Genes regulating membrane-associated E-cadherin and proliferation in adenomatous polyposis coli mutant colon cancer cells: High content siRNA screen
Source: PLoS One. 2020 Oct 15;15(10):e0240746. doi: 10.1371/journal.pone.0240746 (PMC7561197; doi:10.1371/journal.pone.0240746)

S2 Fig. siRNAs with sequence identity to the mir200 family.

A

seed	miRNAs sharing seed
AGTATT	**hsa-miR-200b-3p|hsa-miR-200c-3p|hsa-miR-429**

sense sequence	target gene
GACCTCACCATC**AGTATT**A	BCL9L
TGTCCGAGCTGC**AGTATT**A	ERGIC3
ACAGGGATGTGC**AGTATT**T	NAPSA
GGAGGTATATGC**AGTATT**T	NEK4
GAACGGAAAAGC**AGTATT**A	PHLDB2
CGACTCGGATGC**AGTATT**A	RND1
GCTCCATCCGCC**AGTATT**A	SPDEF
ACATAAAGGTCC**AGTATT**T	TOP1
GGAGGTACCTGC**AGTATT**A	TUBGCP6
GCATAGCCACTC**AGTATT**T	UBE2E3 *
GGAGCACAATCC**AGTATT**T	XPNPEP1

B

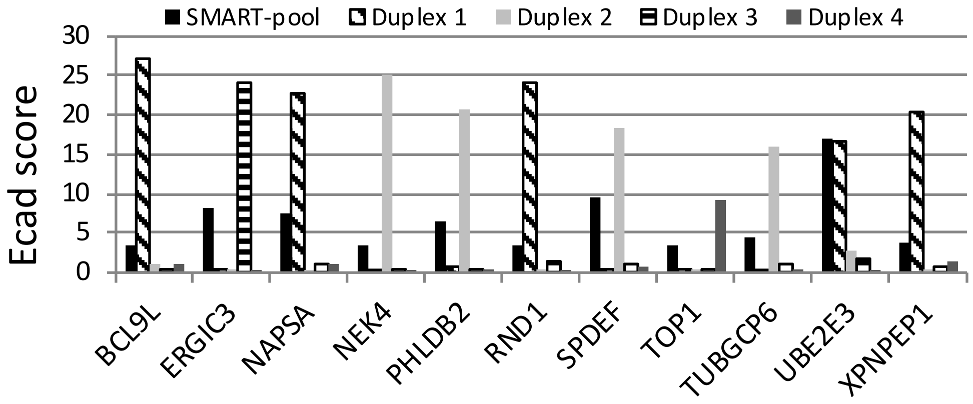

Supplement: S2 Fig — (A) Gene targets with a single siRNA duplex that encodes a miR-200 family seed sequence (see S3, part vi Fig). (B) Dharmacon micro-RNA seed sequence analysis was carried out on the SMARTpool siRNA sequences of 454 genes. siRNAs with sequence identify to the seed sequence on the miR-200 family increased the levels of membrane-associated E-cadherin. These miRNA have a defined role in E-cadherin regulation and therefore any changes with these siRNA are likely caused by a direct effect on miRNAs rather than a specific gene. (PDF) [file pone.0240746.s002.pdf]
