# Supplementary figures and images for: Genes regulating membrane-associated E-cadherin and proliferation in adenomatous polyposis coli mutant colon cancer cells: High content siRNA screen
Source: PLoS One. 2020 Oct 15;15(10):e0240746. doi: 10.1371/journal.pone.0240746 (PMC7561197; doi:10.1371/journal.pone.0240746)

### S3 Fig. Data analysis workflow.

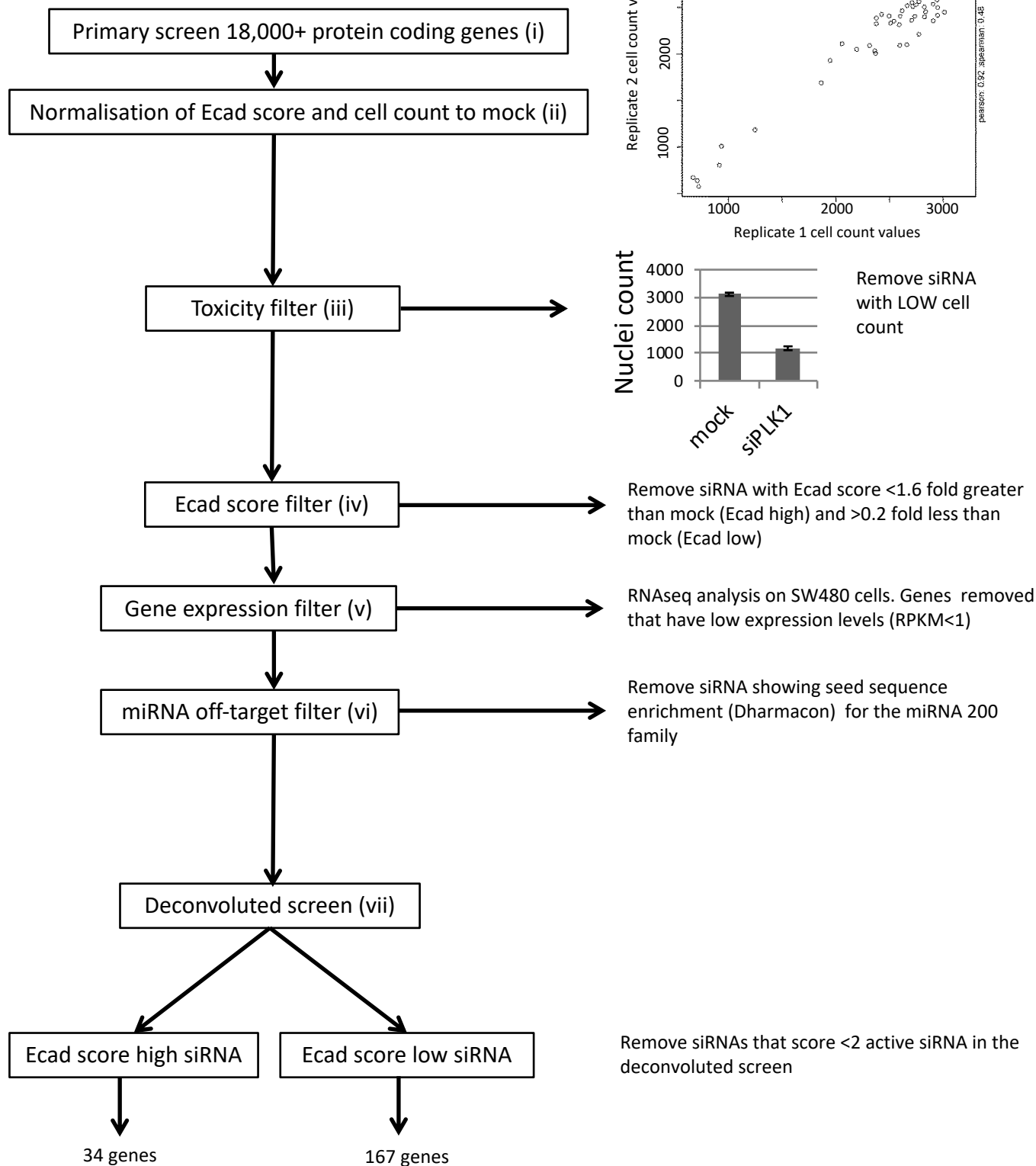

Supplement: S3 Fig — The Dharmacon SMARTpool protein coding library comprised 18120 genes (RefSeq v.27) and was screened in 384 well format, duplicate plates per transfection (i). Raw cell count (total number of cells identified from Hoechst stain/well) and Ecad score were averaged over the duplicate plates for all controls and SMARTpool siRNAs. The total number of mock control wells were averaged per plate (16 wells per primary screen plate and 31 wells per deconvolution screen plate). The raw cell count and Ecad Scores for all SMARTpool siRNAs and the remaining control siRNAs were then normalised to the mock control (from the same plate) (ii). siRNAs were excluded from further analysis based on low cell counts (iii). siPLK1 was used as a toxicity gene control to assess and define cut-off scores for low cell count and to ensure reproducible transfection conditions each transfection. siRNA were binned into the following Cell Viability categories based on cell count; CV1, CV2 and Low Count (LC). CV1: ≥ 0.7 -fold vs mock, CV2: ≥ 0.5 <0.7 -fold vs mock, LC: < 0.5 -fold vs mock. The target cell count per well was set to 3000 and the maximum number of fields was set to 25 to be binned into CV1 category. The minimum number of cells per field was set at 14 and the maximum number of continuous sparse fields (ie fields where there are less than 14 cells) was set to 6. siRNAs in the LC category (i.e <1500 cell count in 25 FOV) were excluded from further analysis. siRNAs were removed from further analysis based on Ecad score (iv). siZEB1 and siCDH1 were used as Ecad Score positive controls to assess and define cut-off values for the high and low Ecad thresholds. siRNAs were binned into the following Ecad Score categories; High (siZEB1 like siRNA): Ecad score 1.6≥ -fold vs mock, NC: Ecad score >0.2, <1.6 –fold vs mock, Low (siCDH1 like siRNA): Ecad score ≤0.2 –fold vs mock. siRNAs were not analysed further if they had an Ecad score in the NC category (v). RNA from SW480 cells was sequenced and anal [file pone.0240746.s003.pdf]

**S4 Fig. Pro-survival and anti-proliferative genes identified in the screen.**

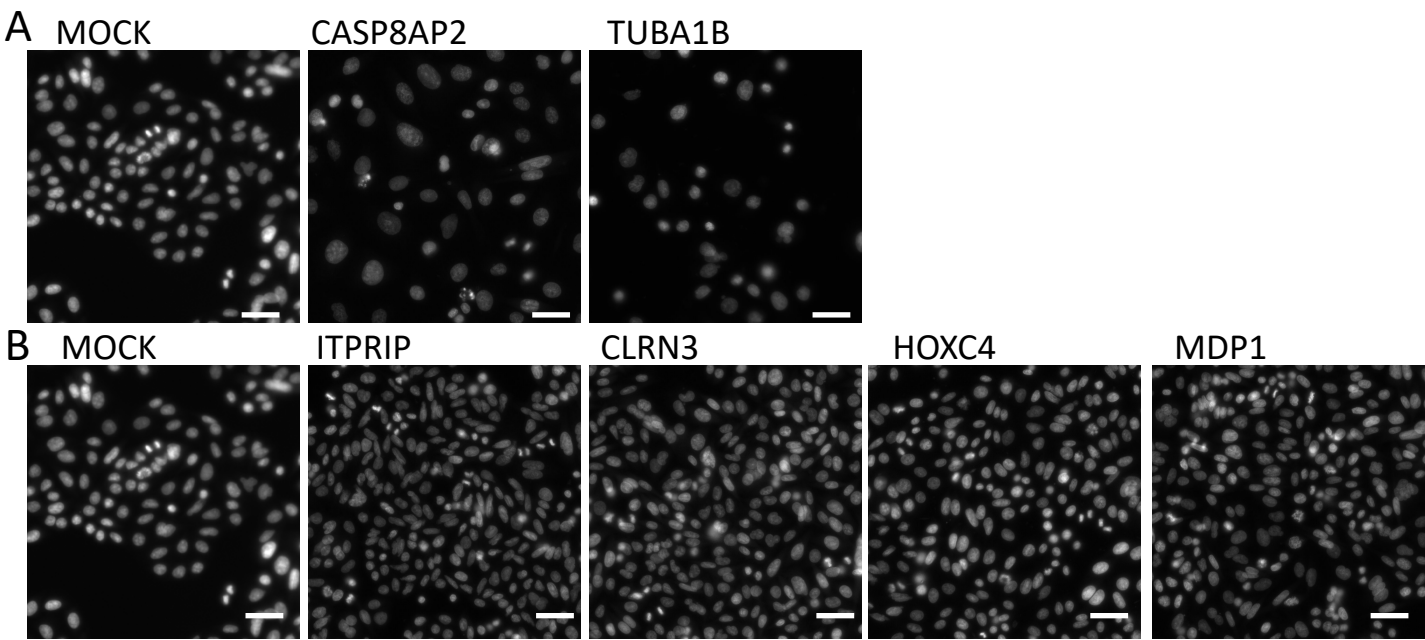

Supplement: S4 Fig — (A) Pro-survival genes. The number of cells/field is reduced when pro-survival genes are knocked down. Representative images from the screen of Hoechst staining 72 hours post transfection of siRNAs for mock, CASP8AP2 and TUBA1B. Scale bar; 50 μM. (B) Anti-proliferative genes. The number of cells/field is increased when anti-proliferative genes are knocked down. Representative images from the screen of Hoechst staining 72 hours post transfection of siRNAs for mock, ITPRIP, CLRN3, HOXC4 and MDP1. Scale bar; 50 μM. (PDF) [file pone.0240746.s004.pdf]

# S5 Fig. SNX27 is a negative regulator of membrane associated E-cadherin.

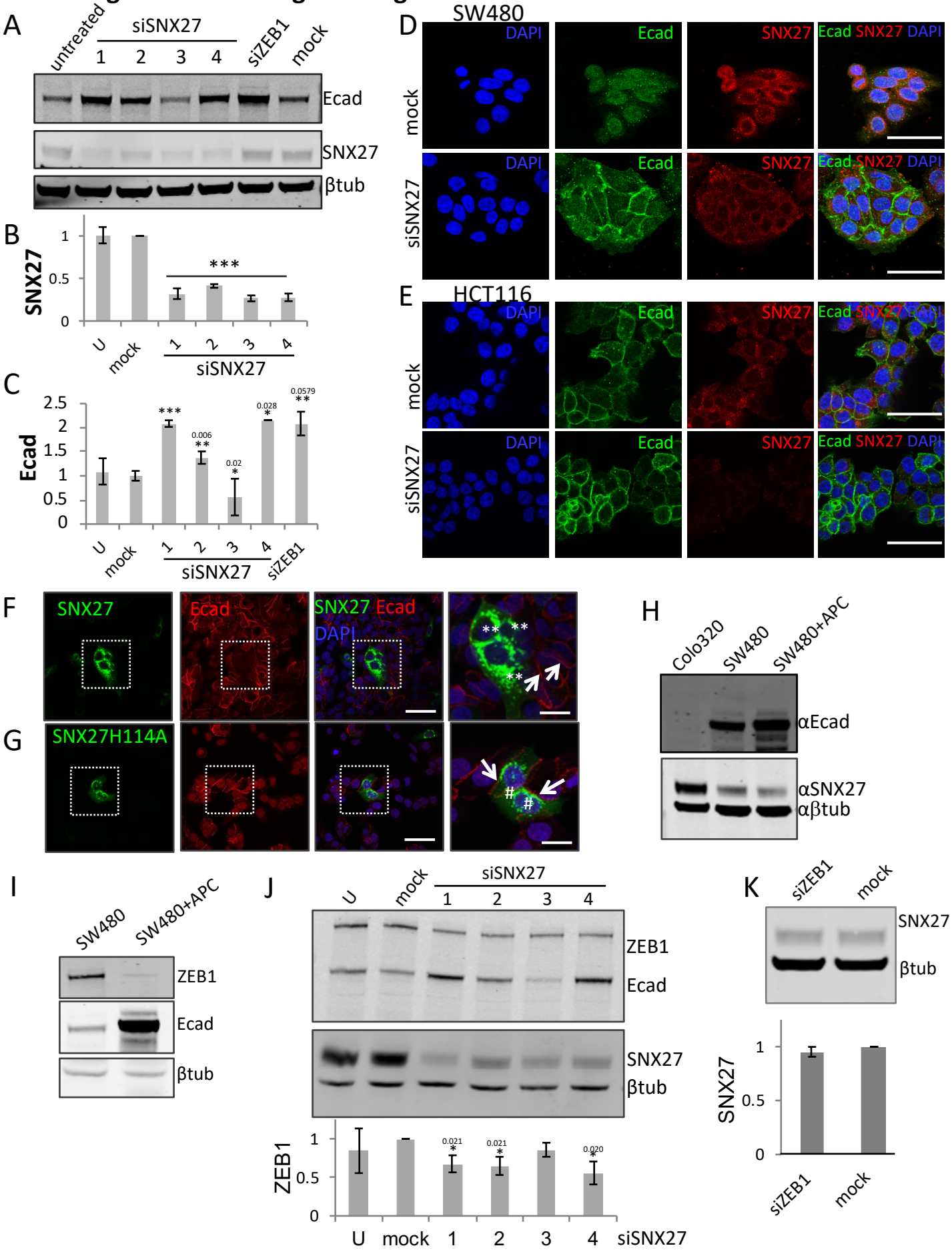

Supplement: S5 Fig — (A) Whole cell lysate immunoblot analysis siSNX27 knockdown in SW480 cells. Cells were transfected using identical siRNA oligo sequences that were used in the screen for SNX27 or ZEB1 (SMARTpool) and protein levels were assessed 72 hours later. The blot was probed with antibodies against E-cadherin, SNX27 and β-tubulin (loading control) and is representative of 4 individual experiments. (B and C) Quantification of SNX27 (B) and E-cadherin protein (C) levels upon siSNX27 knockdown in SW480 cells (n = 4). Protein levels were determined using densitometry against the loading control β-tubulin and displayed as the mean ± SEM For SNX27 (B) ***p<0.001 for all samples vs mock control; for E-cadherin (C) ***p<0.001 for SNX27 si #1; **p<0.01 (p = 0.006 for SNX27si #2, and p = 0.005796 for ZEB1 si), *p<0.05 (p = 0.02 and p = 0.028 for SNX27si #3 and 4, respectively), for all samples vs mock control, paired one-tailed Student’s t-test. (D and E) SNX27 depletion promotes junctional E-cadherin in SW480 cells (D) and HCT116 cells (E). E-cadherin (Ecad) (green), SNX27 (red) and nuclei (DAPI) (blue). Scale bar 50μm. (F and G) SNX27 regulates cell adhesion through an interaction in the SNX27PDZ domain. SNX27-eGFP expression disrupts junctional E-cadherin in SW480+APC cells (F) but PDZ-domain mutant, SNX27-H114A-eGFP expression does not (G). Cell contacts are indicated by arrows. Junctional staining is absent in SNX27-eGFP expressing cells ** (F) but are intact in SNX27-H114A-eGFP expressing cells # (G). SNX27-eGFP and SNX27-H114A-eGFP (green), E-cadherin (red), nuclei (DAPI) (blue). Scale bar 50μm, left hand panels and 80μm, enlarged inset, right hand panels. (H) Whole cell lysate immunoblot analysis of SNX27 and E-cadherin levels in Colo320, SW480 and SW480+APC cells. β-tubulin serves as a loading control. (I) Immunoblot analysis of ZEB1, E-cadherin and β-tubulin in SW480 and SW480+APC cells. Shown are cropped blots, representative of three independent experiments. (J) ZEB1 express [file pone.0240746.s005.pdf]

**S6 Fig. Post transcriptional regulation of E-cadherin by MMP14 and MMP19**

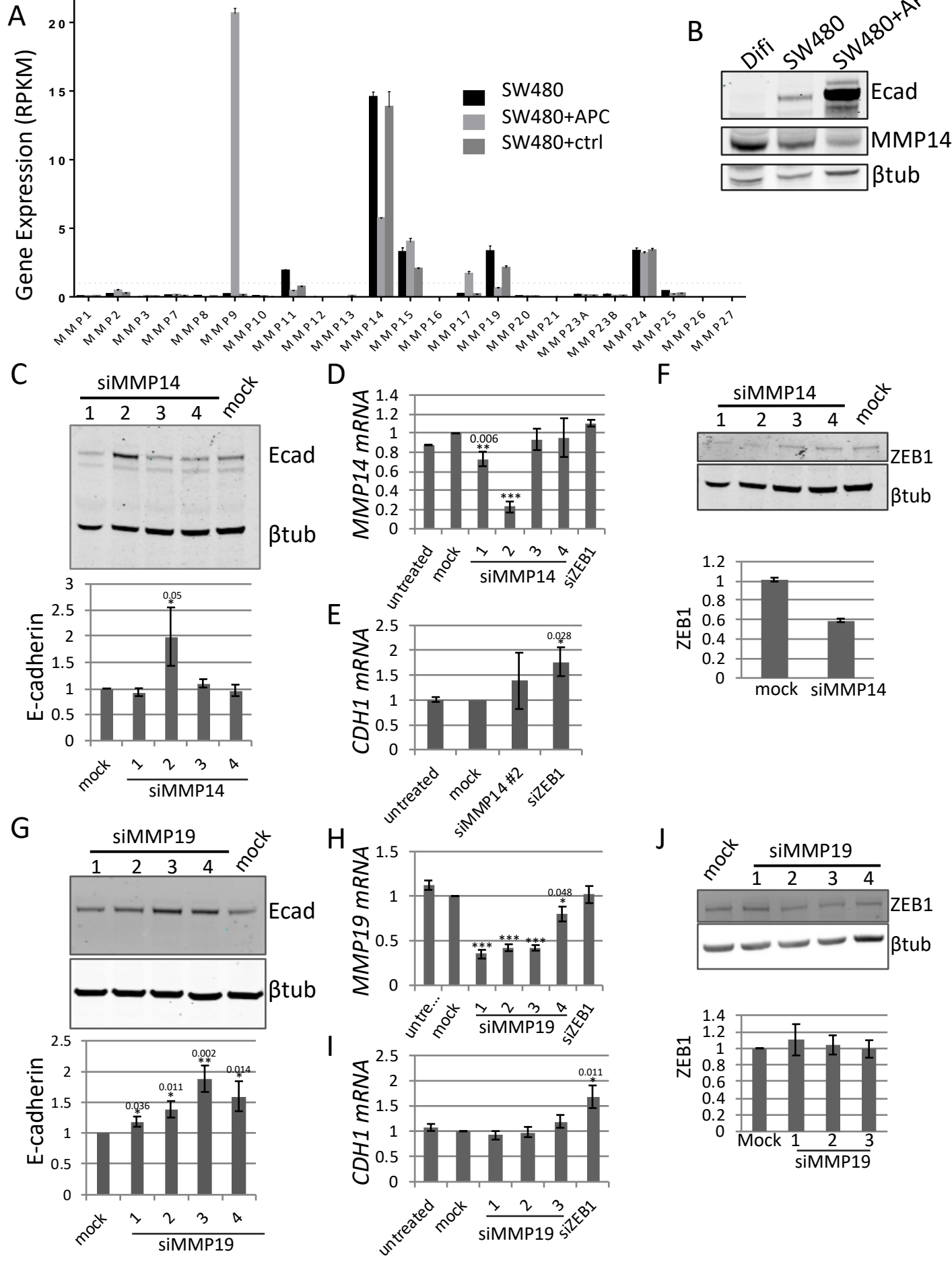

Supplement: S6 Fig — (A) Differential RNAseq analysis of MMP gene expression for SW480, SW480+APC and SW480 +control (SW480+ctrl) cells. Shown is the MEAN ± Std Dev of triplicate samples. (B) Whole cell lysate immunoblot analysis of MMP14 and E-cadherin in Difi, SW480 and SW480+APC cells. β-tubulin serves as a loading control. (C) siMMP14 knockdown (duplex#2) in SW480 cells promotes E-cadherin. E-cadherin immunoblot analysis from cells transfected with siMMP14 duplexes for 72 h. Quantification is shown in the plot below, Mean± SEM (n = 4, *p = 0.05, one-tailed unpaired Student’s t-test vs mock control). (D) MMP14 mRNA expression from SW480 cells transfected with siRNAs #1–4 or siZEB1 (SMARTpool) for 72 hours. Shown is MEAN ± SEM (n = 4), **p<0.01 (p = 0.006), ***p<0.001), one-tailed unpaired Student’s t-test vs mock control. Note only duplex #2 results in depletion of MMP14. (E) CDH1 mRNA expression from SW480 cells transfected with siMMP14 #2 or siZEB1 (SMARTpool) for 72 hours. Shown is mean ± SEM (n = 4), *p<0.05 (p = 0.028), one-tailed paired Student’s t-test vs mock control. (F) Whole cell lysis analysis of ZEB1 expression after knockdown of MMP14 #1–4. Quantification of ZEB1 protein levels (Mean ± SD (n = 2)) is shown below the representative blot. (G) siMMP19 knockdown in SW480 cells promotes E-cadherin. E-cadherin immunoblot analysis from cells transfected with siMMP19 duplexes for 72 h. Cells were harvested 72 hours post-transfection and whole cells lysates probed with antibodies against E-cadherin and β-tubulin. Quantification is shown in the plot below. Mean± SEM (n = 5) *p<0.05, **P<0.005 (exact p values are indicated) one-tailed unpaired Student’s t-test vs mock control. (H) MMP19 mRNA expression from SW480 cells transfected with siMMP duplexes #1–4 or siZEB1 (SMARTpool) for 72 hours. Shown is MEAN ± SEM (n = 4) *p<0.05 (p = 0.048), ***p<0.001 one-tailed paired Student’s t-test vs mock control. (I) CDH1 mRNA expression from SW480 cells transfected with siMMP19 duplexes #1–4 [file pone.0240746.s006.pdf]
