# Supplementary material for: Genes regulating membrane-associated E-cadherin and proliferation in adenomatous polyposis coli mutant colon cancer cells: High content siRNA screen
Source: PLoS One. 2020 Oct 15;15(10):e0240746. doi: 10.1371/journal.pone.0240746 (PMC7561197; doi:10.1371/journal.pone.0240746)

# S1 raw images

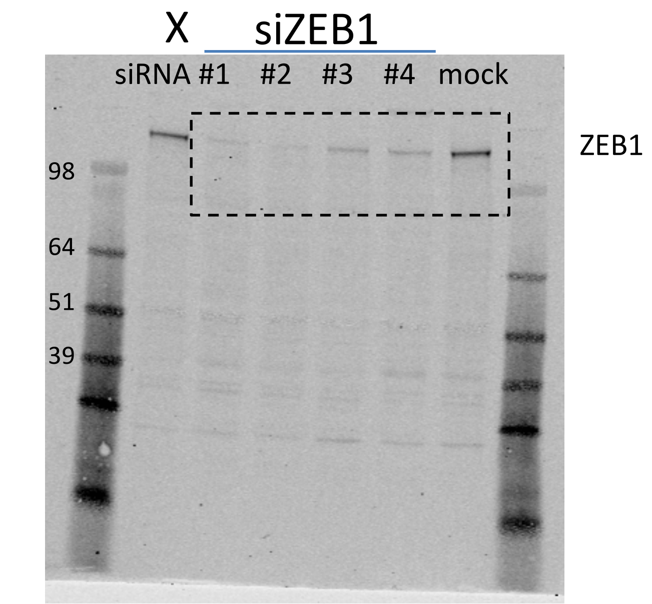


Probed with antibodies to ZEB1

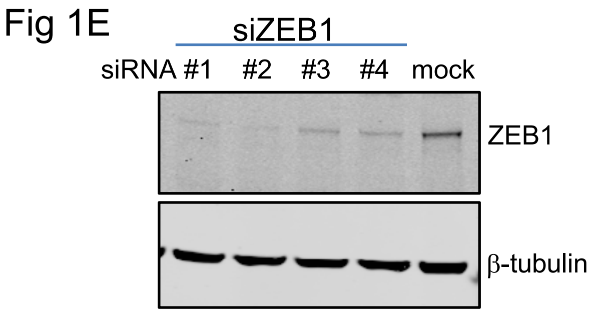


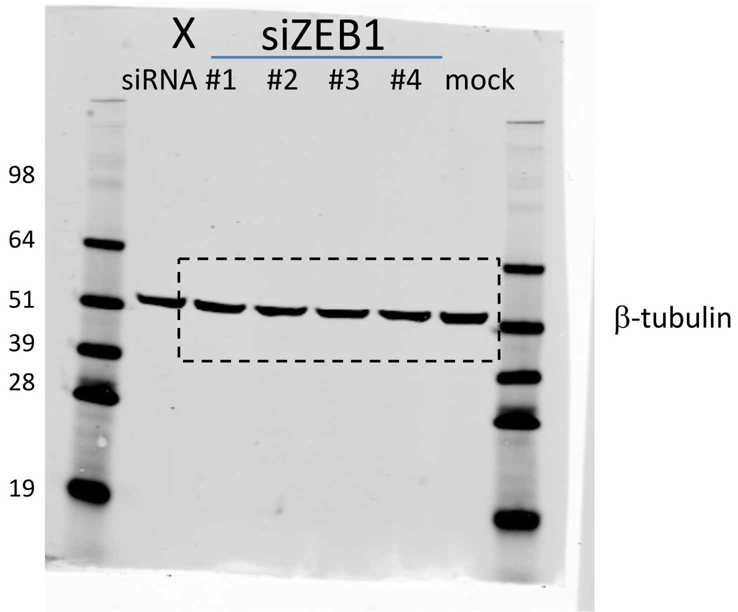


RE-probed with antibodies to β-tubulin

Original blots from Fig 1E
SW480 cells were transfected with individual siRNAs from the ZEB1 SMARTpool for 72 hours. The blots were probed with antibodies as indicated.

# S1 raw images

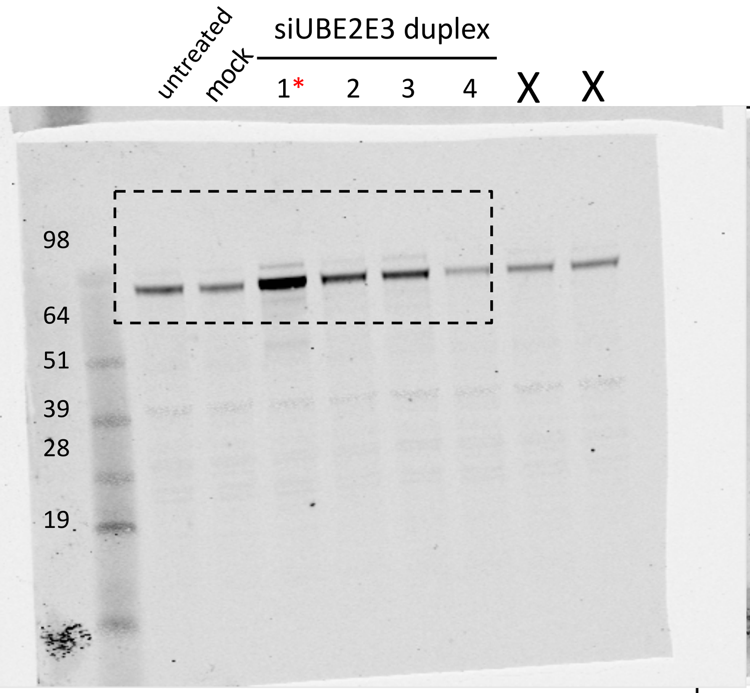


Probed with antibodies to E-cadherin

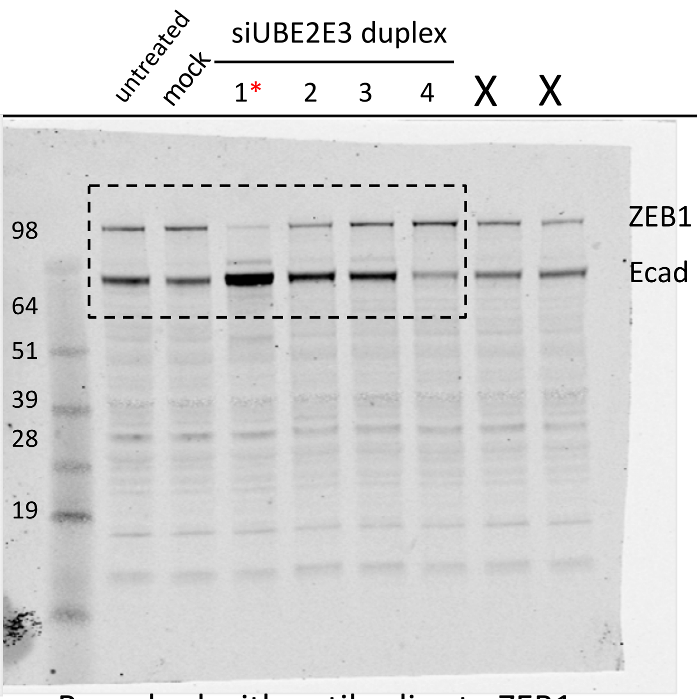


Reprobed with antibodies to ZEB1

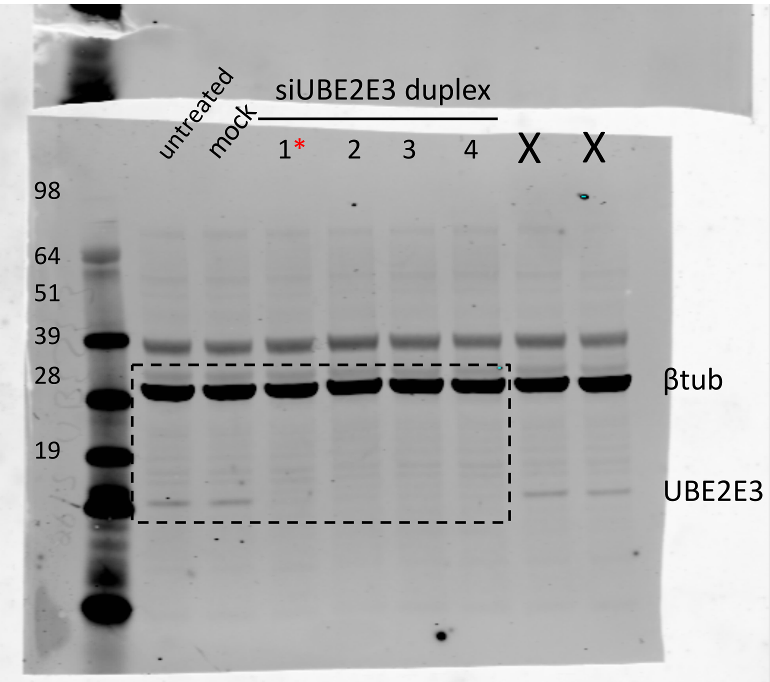


βtubulin and UBE2E3

Fig 3A

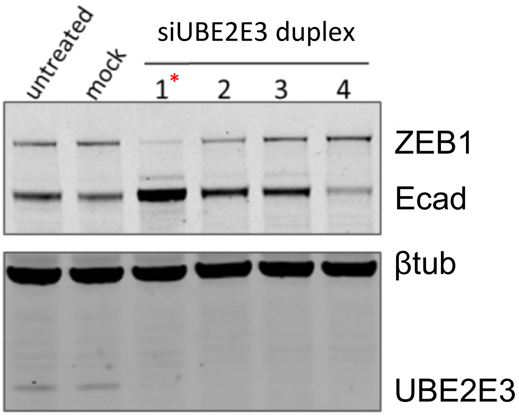


Original blots from Fig 3A

SW480 cells were transfected with individual siRNAs from the UBE2E3 SMARTpool for 72 hours. The blots were probed with antibodies as indicated.

# S1 raw images

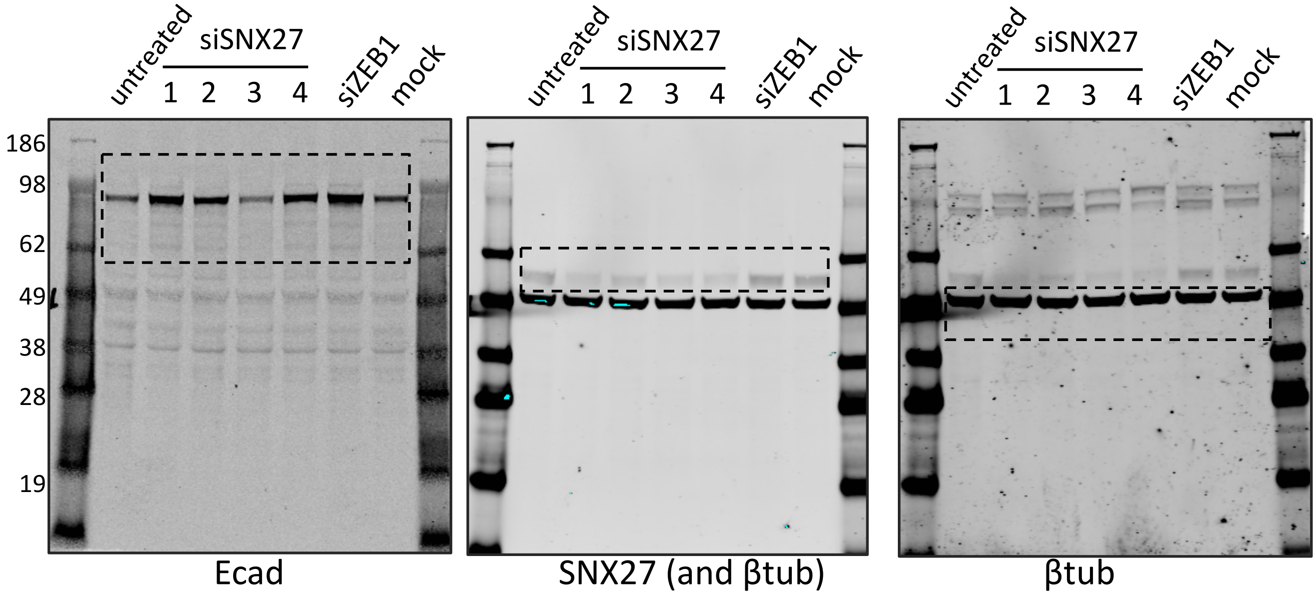


Original blots from S5A Fig
SW480 cells were transfected using identical siRNA oligo sequences that were used in the screen for SNX27 or ZEB1 (SMARTpool) and protein levels were assessed 72 hours later. The blots were probed with antibodies as indicated.

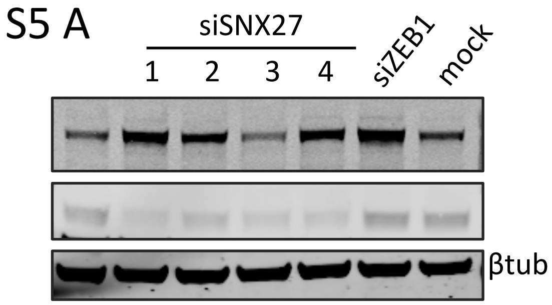

# S1 raw images

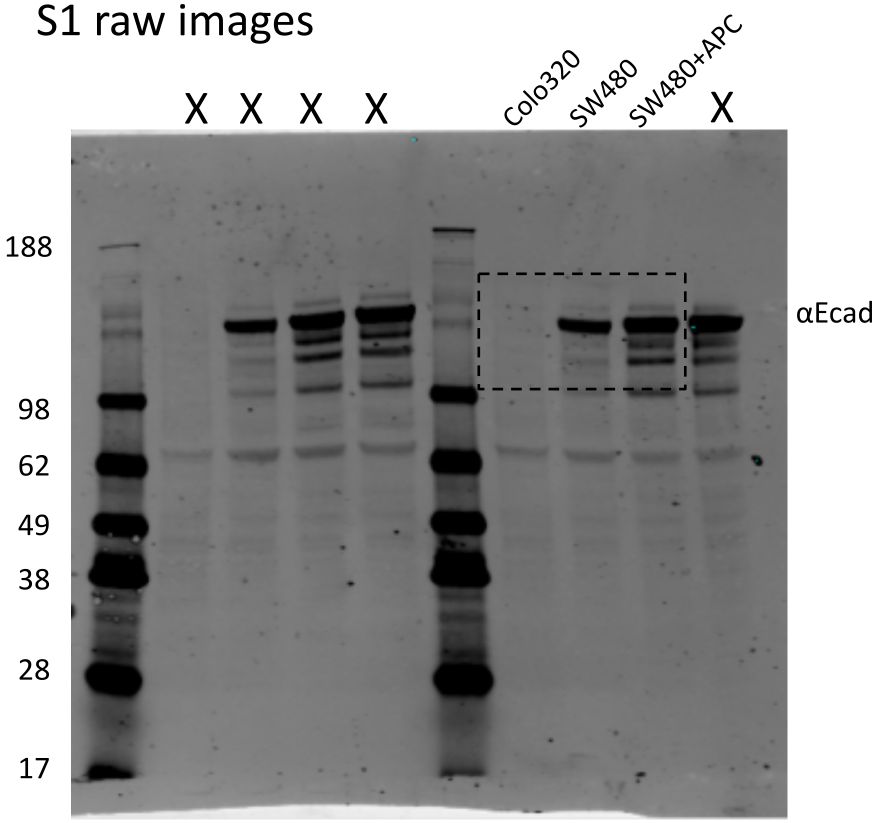


Ecadherin

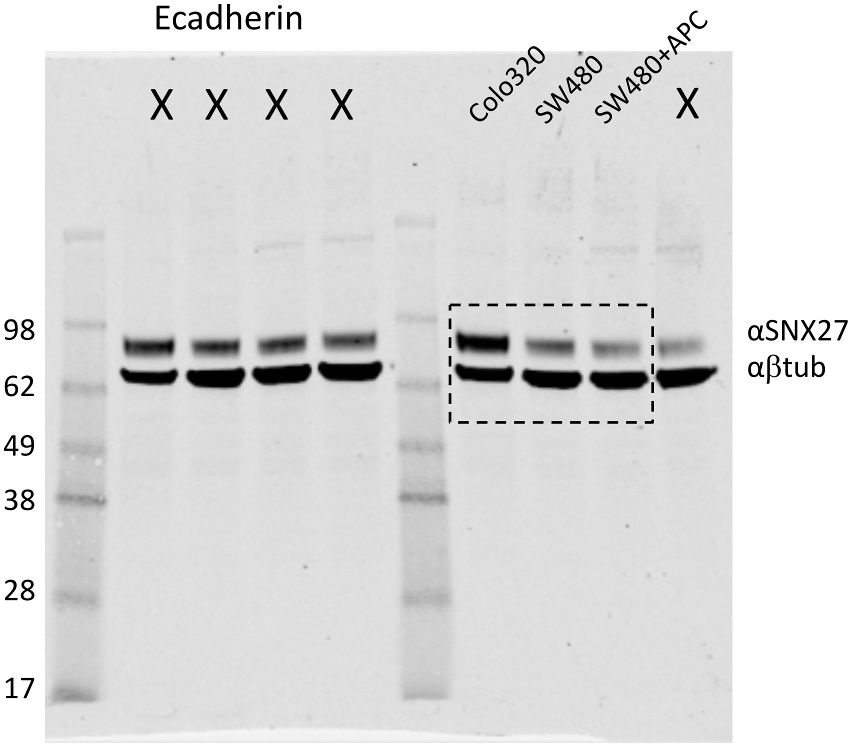


SNX27 and βtub

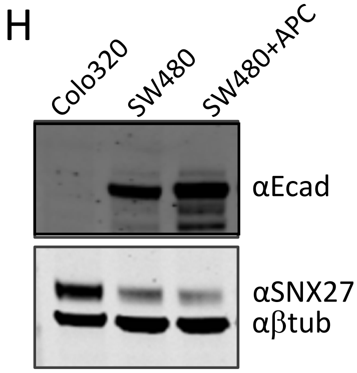


Original blots from S5H Fig

Whole cell lysate immunoblot analysis of SNX27 and E-cadherin levels in Colo320, SW480 and SW480+APC cells. β-tubulin serves as a loading control.

# S1 raw images

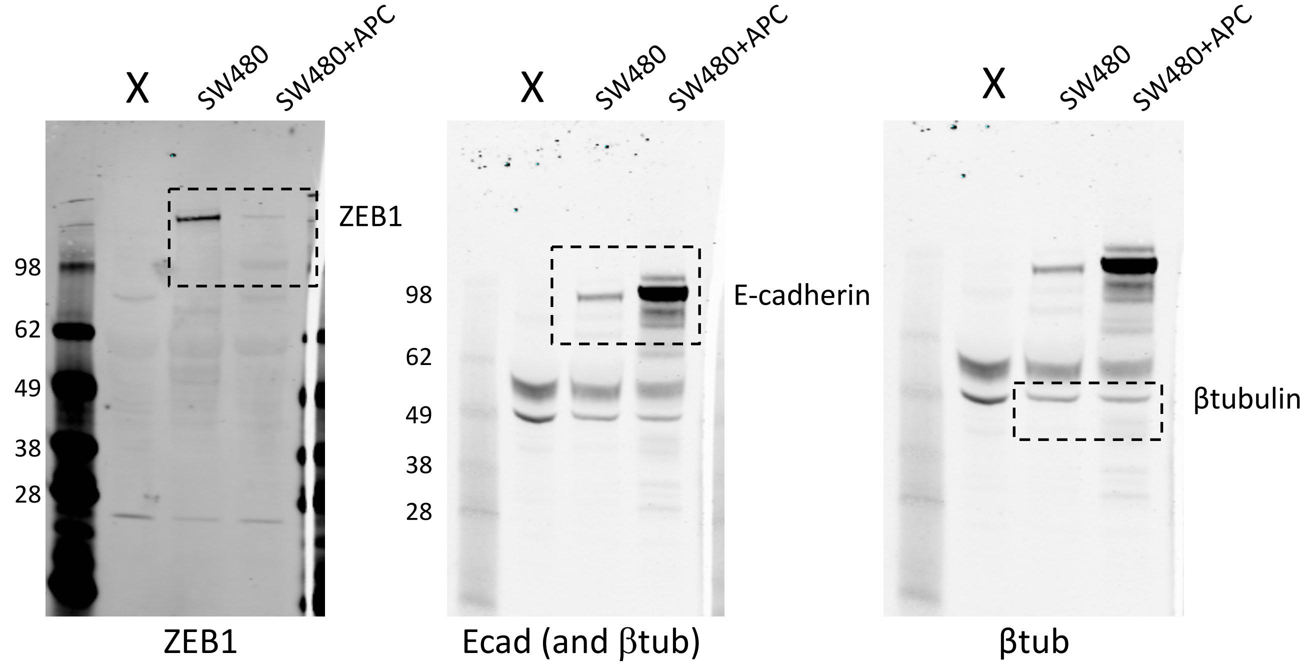


Original blots from S5I Fig
Immunoblot analysis of ZEB1, E-cadherin and β-tubulin in SW480 and SW480+APC cells.

# S5 I

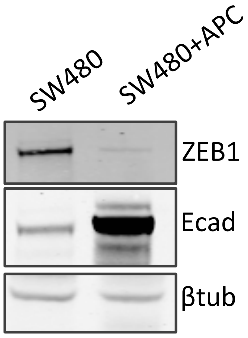

# S1 raw images

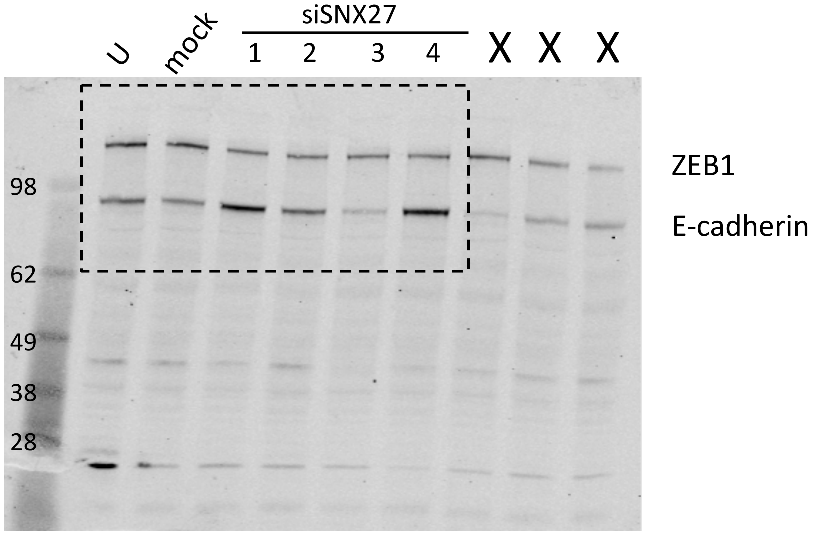


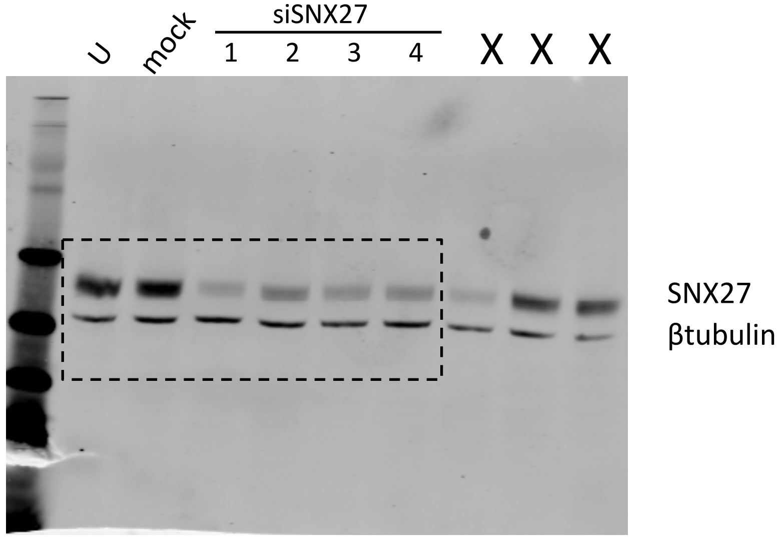


Original blots from S5J Fig

ZEB1 expression upon siSNX27 knockdown in SW480 cells. Cells were transfected with siSNX27 duplexes and harvested 72 hours post-transfection. Whole cell lysates were probed with antibodies against E-cadherin, ZEB1 SNX27 and β-tubulin (loading control).

# S5 J

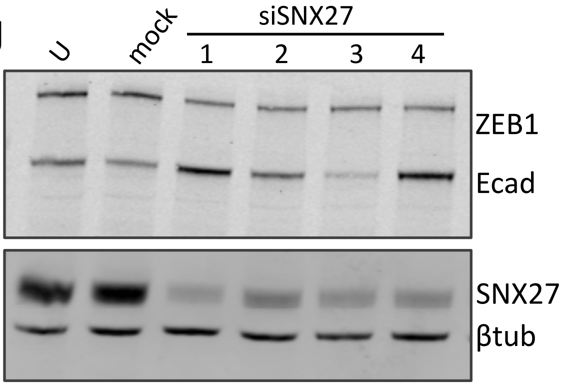

# S1 raw images

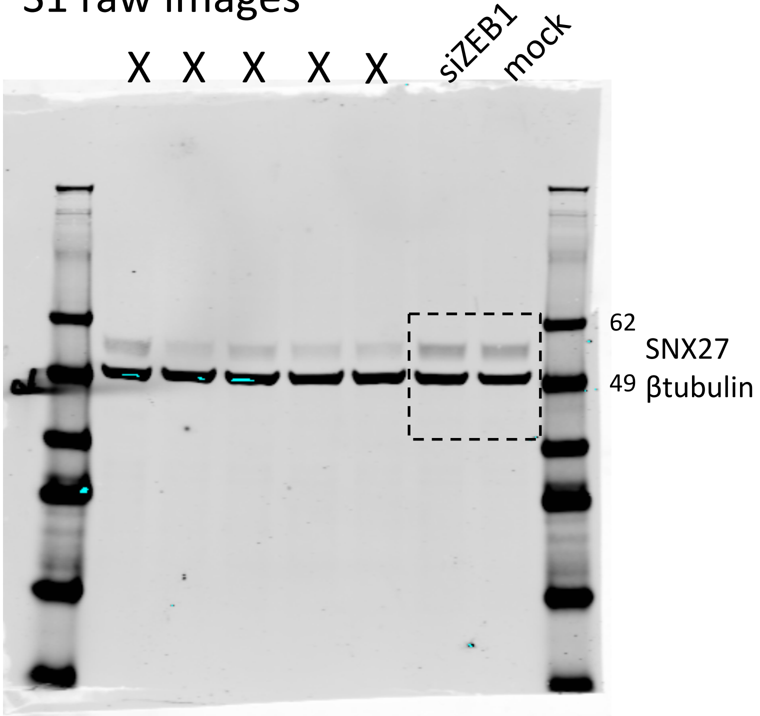


SNX27 and βtub

## S5 K

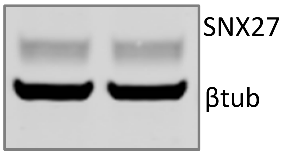


Original blots from S5K Fig
SW480 cells were transfected with siZEB1 (SMARTpool) and protein levels were assessed 72 hours later. The blot was probed with SNX27 and β-tubulin (loading control) antibodies.

# S1 raw images

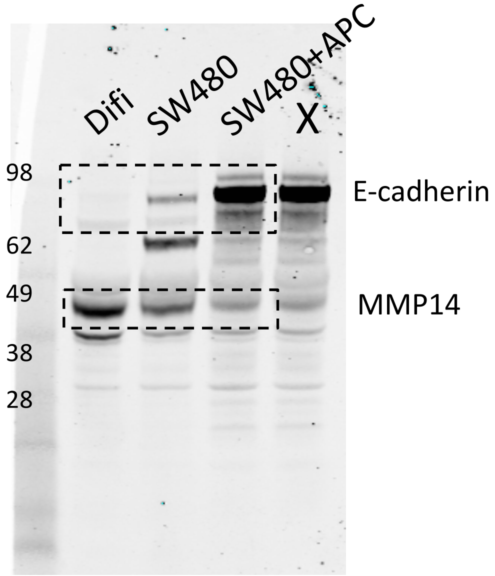


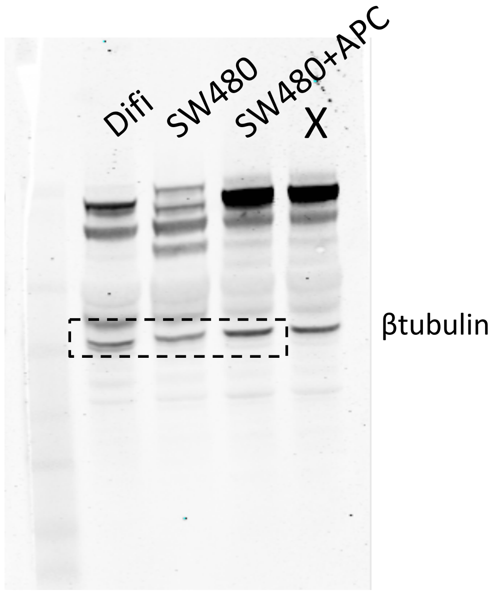


S6 B

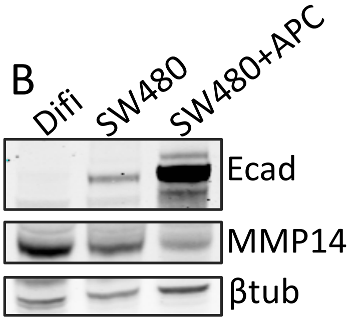


Original blots from S6B Fig

Whole cell lysate immunoblot analysis of MMP14 and E-cadherin in Difi, SW480 and SW480+APC cells (left hand side). β-tubulin serves as a loading control. (right hand side)

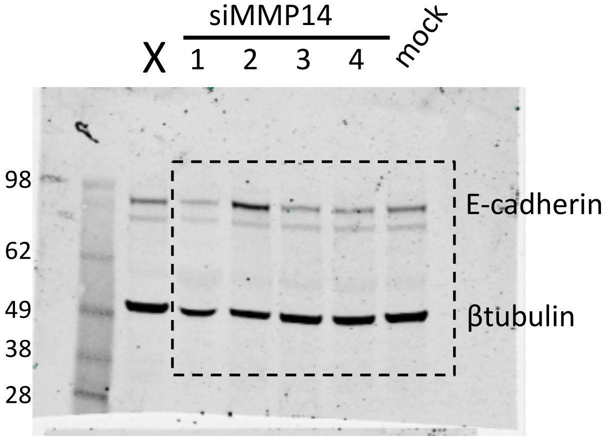


Ecad and βtubulin

S6 C

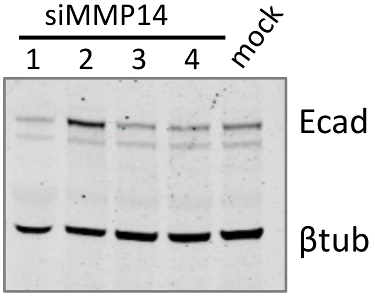


Original blots from S6C Fig

E-cadherin immunoblot analysis from cells transfected with siMMP14 duplexes. Cells were harvested 72 hours post-transfection and whole cell lysates probed with antibodies against E-cadherin and β-tubulin.

# S1 raw images

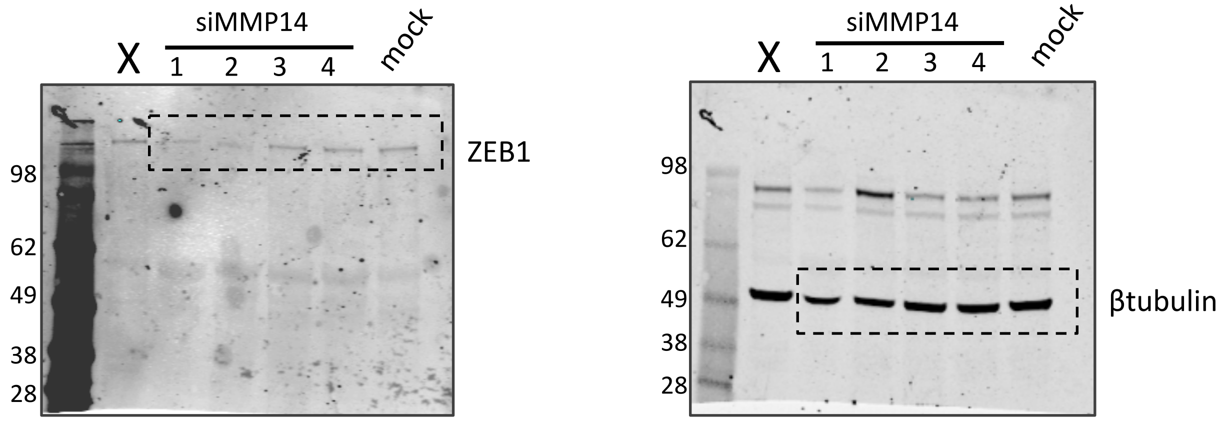


Original blots from S6F Fig

ZEB1 immunoblot analysis from cells transfected with siMMP14 duplexes. Cells were harvested 72 hours post-transfection and whole cell lysates probed with antibodies against ZEB1 (left) and β-tubulin (right).

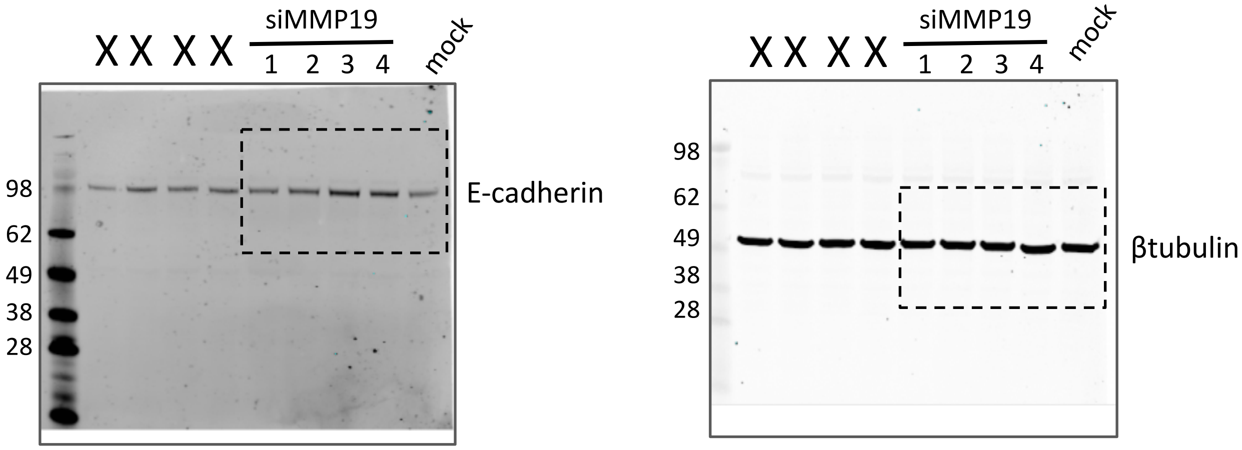


Original blots from S6G Fig

E-cadherin immunoblot analysis from cells transfected with siMMP19 duplexes. Cells were harvested 72 hours post-transfection and whole cells lysates probed with antibodies against E-cadherin (left) and β-tubulin (right).

F

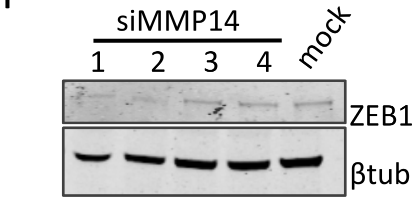


G

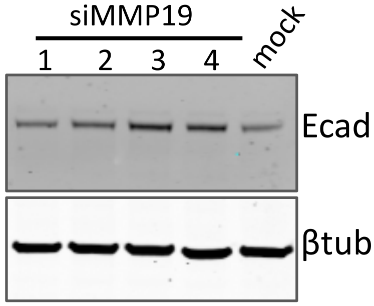

# S1 raw images

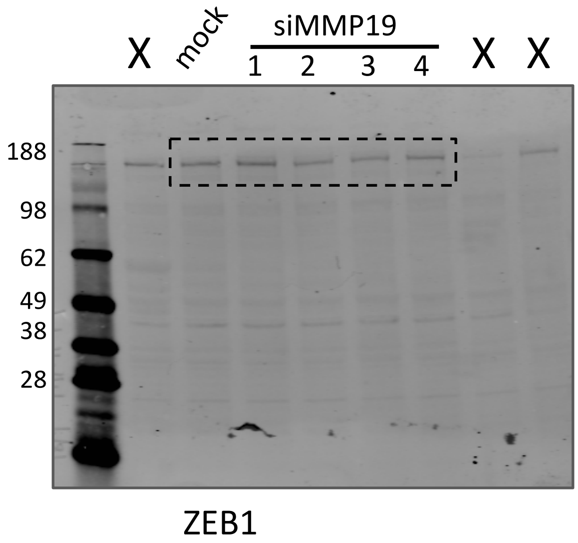


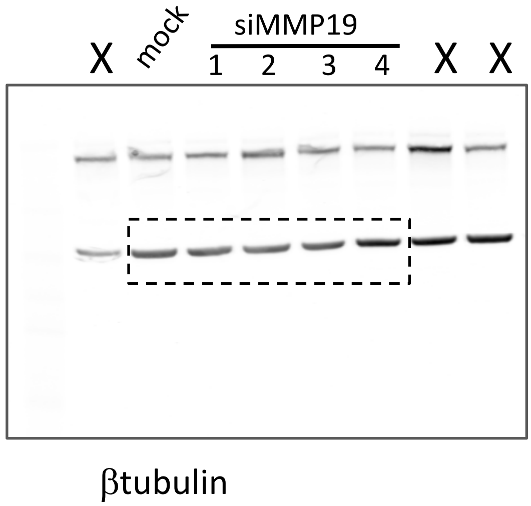


Original blots from S6J Fig

ZEB1 immunoblot analysis from cells transfected with siMMP19 duplexes. Cells were harvested 72 hours post-transfection and whole cells lysates probed with antibodies against ZEB1 (left) and β-tubulin (right).

S6 J

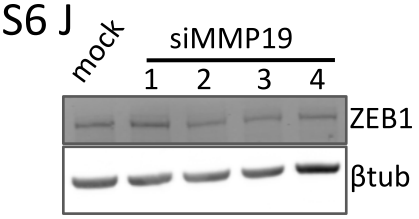

Supplement: S1 Raw images — (PDF) [file pone.0240746.s007.pdf]
