# Supplementary material for: Genes regulating membrane-associated E-cadherin and proliferation in adenomatous polyposis coli mutant colon cancer cells: High content siRNA screen
Source: PLoS One. 2020 Oct 15;15(10):e0240746. doi: 10.1371/journal.pone.0240746 (PMC7561197; doi:10.1371/journal.pone.0240746)
Supplement: S1 File — (DOCX) [file pone.0240746.s011.docx]

**Supporting Information Reference List**

1. King LE, Love CG, Sieber OM, Faux MC, Burgess AW. Differential RNA-seq analysis comparing APC-defective and APC-restored SW480 colorectal cancer cells. Genomics data. 2016;7:293-6.
2. Smits R, Ruiz P, Diaz-Cano S, Luz A, Jagmohan-Changur S, Breukel C, et al. E-cadherin and adenomatous polyposis coli mutations are synergistic in intestinal tumor initiation in mice. Gastroenterology. 2000;119(4):1045-53.
