# Supplementary material for: Genes regulating membrane-associated E-cadherin and proliferation in adenomatous polyposis coli mutant colon cancer cells: High content siRNA screen
Source: PLoS One. 2020 Oct 15;15(10):e0240746. doi: 10.1371/journal.pone.0240746 (PMC7561197; doi:10.1371/journal.pone.0240746)
Supplement: Attachment — Submitted filename: Response to Reviewers.docx [file pone.0240746.s013.docx]

Chunming Liu
Academic Editor
PLOS ONE

Dear Professor Liu,

RE: PONE-D-20-23014
Genes regulating membrane-associated E-cadherin and proliferation in adenomatous polyposis coli mutant colon cancer cells: High content siRNA screen

Thank you for your invitation to submit a revised version of our manuscript. We are pleased to see that both reviewers agree that ‘All comments have been addressed’ and that all other requirements have been resolved. We have addressed the remaining concern raised by Reviewer 1 in our detailed response below:

6. Review Comments to the Author

Reviewer #1: While all comments have been addressed, there is still concern about the use of thioglycerol in the cell culture media for the SW480 cells. The authors state that it is needed for proliferation. While thio is used in some primary cell culture media, it should not be required for culturing the highly aggressive SW-480 cells. The concern here is that there is something inherently wrong with these cells that require thioglycerol to grow, which may also influence the results described in the manuscript.

*Response:* We have routinely cultured colon cancer cell lines in RPMI supplemented with 10%FCS, insulin, thioglycerol and hydrocortisone. We have found that this provides consistent growth conditions for a range of cell lines and have used this media for culturing the SW480 cells in this study. We understand the reviewer's concern for a robust cell line such as SW480 cells, but we have also grown the SW480 cells in other media such as DME plus 10% FCS and have not observed a difference in growth rate. Furthermore, the various controls that we used in the experiments in the manuscript do not show adverse effects on proliferation or other growth characteristics. We do not believe that there is something inherently wrong with these cells that require thioglycerol to grow.

We have included the following in the revised manuscript:

‘Thioglycerol is used in culture media to stimulate proliferation and we have found that this provides consistent growth conditions, but the cells do not require thioglycerol for their growth.’

We trust that our manuscript is now suitable for publication in PLOS ONE.

Yours sincerely,

Maree Faux

Dr Maree Faux,

The Walter and Eliza Hall Institute of Medical Research,

1G Royal Parade, Parkville, VICTORIA 3051

Australia

Email: [faux@wehi.edu.au](mailto:faux@wehi.edu.au)
